# A mobile approach-avoidance task

**DOI:** 10.3758/s13428-020-01379-3

**Published:** 2020-03-16

**Authors:** Hilmar G. Zech, Mark Rotteveel, Wilco W. van Dijk, Lotte F. van Dillen

**Affiliations:** 1 Leiden University, Leiden, the Netherlands; 2 University of Amsterdam, Amsterdam, the Netherlands

**Keywords:** AAT, approach-avoidance, mobile, reaction times, force

## Abstract

Approach and avoidance tendencies have helped explain phenomena as diverse as addiction (Mogg, Field, & Bradley, 2005), phobia (Rinck & Becker, 2007), and intergroup discrimination (Bianchi, Carnaghi, & Shamloo, 2018; Degner, Essien, & Reichardt, 2016). When the original approach-avoidance task (AAT; Solarz, 1960) that measures these tendencies was redesigned to run on regular desktop computers, it made the task much more flexible but also sacrificed some important behavioral properties of the original task—most notably its reliance on physical distance change (Chen & Bargh, 1999). Here, we present a new, mobile version of the AAT that runs entirely on smartphones and combines the flexibility of modern tasks with the behavioral properties of the original AAT. In addition, it can easily be deployed in the field and, next to traditional reaction time measurements, includes the novel measurement of response force. In two studies, we demonstrate that the mobile AAT can reliably measure known approach-avoidance tendencies toward happy and angry faces both in the laboratory and in the field.

## A mobile approach-avoidance task

Automatic approach-avoidance tendencies play a role in many of today’s societal problems. Addictions, for example, drive people to approach harmful substances (Mogg, Field, & Bradley, 2005), whereas phobias cause them to avoid things that are harmless (Rinck & Becker, 2007). Prejudice drives people to avoid individuals from another group (Bianchi, Carnaghi, & Shamloo, 2018) and overeating might involve people approaching food beyond fulfilling their caloric needs. Such approach-avoidance tendencies are difficult to measure using self-reports as they can influence behavior within split-seconds after a stimulus is encountered. Therefore, researchers have developed approach-avoidance tasks (AATs) that assess these tendencies behaviorally (Solarz, 1960). When the original AAT (Solarz, 1960) was redesigned to run on personal computers (Chen & Bargh, 1999; Rinck & Becker, 2007), this greatly increased the flexibility of the task and facilitated its application across many different research areas. Yet, although computerized AATs are more flexible than the original, custom-build AAT, they do not lend themselves well to field research, as they require specialized hardware that is not available in most households. To overcome this limitation, we developed a smartphone-based version of the AAT, which combines the behavioral properties of the original AAT with the flexibility and field-readiness provided by smartphones. In addition to these features, the mobile AAT introduces a novel measurement of response force, which could further inform approach-avoidance theory. To illustrate the positive features of the mobile AAT, we will, in the following, first provide a brief overview of the original and of modern, computerized AATs.

In the original AAT, Solarz (1960) presented participants with positive and negative word cards, and asked them to pull some cards toward themselves and to push other cards away from themselves. He measured how quickly participants initiated their responses and found that they were faster when they had to approach positive stimuli (e.g., pull “happy”) or avoid negative stimuli (e.g., push “sad”) compared to when these instructions were reversed (e.g., push “happy” or pull “sad”). His findings, therefore, for the first time, indicated that positive stimuli activate approach tendencies and negative stimuli activate avoidance tendencies. The original AAT measured approach and avoidance in their literal sense, “approach refer[ring] to decreasing, and avoidance to increasing the physical distance between the self and the outside world” (Koch, Holland, Hengstler, & van Knippenberg, 2016, p. 549; see also, Stins et al., 2011). Yet, its custom design also made it difficult for other researchers to set up the AAT and the task was not widely used in the following decades (for an exception, see Angel, 1973).

Computerized AATs, on the other hand, run on regular personal computers and can easily be set up by most researchers. They accomplish this by replacing the physical distance change in Solarz’ design with virtual or suggested distance change. In the joystick AAT, for example, distance change is suggested by a zooming effect, while participants pull or push joysticks to approach and avoid stimuli (Rinck & Becker, 2007). In the manikin AAT—another prominent version of the task—distance change is suggested by moving a manikin toward or away from a stimulus, while participants interact with joysticks, computer mice, or keyboards (Markman & Brendl, 2005). These changes allow computerized AATs to run on personal computers, which led to an exponential increase of lab-based AAT research over the last decades (Eder, Elliot, & Harmon-Jones, 2013).

While computerized AATs made the AAT more accessible than the original task, they still have one downside: Their reliance on stationary hardware makes it difficult to use them in the field. Consequently, most existing AAT research has focused on stable approach-avoidance tendencies. Research on dynamic tendencies, on the other hand, for example towards food, has so far yielded inconsistent findings (for a summary, see Meule et al., 2019). It is, however, plausible that such dynamic approach-avoidance tendencies exist. Hofmann, Baumeister, Förster, and Vohs (2012), for example, found that people’s self-reported desires, such as the desire for alcohol or food, fluctuate significantly across times of day and different social settings. Computerized AATs require stationary hardware and do not lend themselves well to the study of such temporal and context-based fluctuations.

**Fig. 1:**
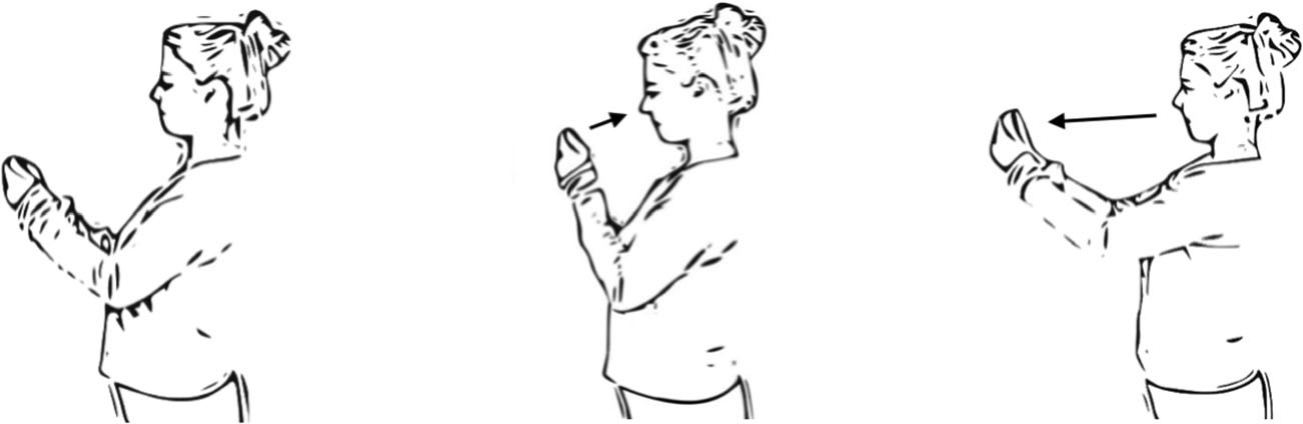
Movements in mobile AAT. Arm position between trials (left), after an approach movement (middle), and after an avoidance movement (right).

To provide a task that can measure approach-avoidance tendencies dynamically, we introduce a new, mobile AAT which can easily be used in the field. The mobile AAT can be downloaded as an app and runs on regular smartphones. Stimuli are presented on the smartphone screen and—similar to the original AAT (Solarz, 1960)—participants naturally approach stimuli by pulling the phone toward themselves and avoid stimuli by pushing the phone away from themselves (see Fig. 1). In this operationalization, the mobile AAT differs from computerized AATs and also from other recently developed mobile AATs (further described in the discussion section).

The mobile AAT’s reliance on natural approach and avoidance movements has two advantages: First, it naturally aligns the mobile AAT with prominent approach-avoidance theories, which—although they disagree about the exact nature of approach-avoidance tendencies—agree that approach tendencies drive people to decrease the distance to a stimulus and avoidance tendencies drive people to increase the distance to a stimulus (Krieglmeyer, De Houwer, & Deutsch, 2013; Eder & Rothermund, 2008). Second, natural distance change helps to disambiguate responses. In most computerized AATs, distance change is suggested virtually, for example, by explicitly labeled responses (Chen & Bargh, 1999), changes in stimulus size (Rinck & Becker, 2007), or movements of an avatar toward or away from the stimulus (De Houwer, Crombez, Baeyens, & Hermans, 2001; Rougier et al., 2018). These AATs require such guiding principles as, in their absence, responses can become ambiguous and approach-avoidance effects can become unreliable or even revert completely (Lavender & Hommel, 2007; Markman & Brendl, 2005; Seibt, Neumann, Nussinson, & Strack, 2008; Stins et al., 2011). The mobile AAT does not require such virtual guidance, as pulling and pushing the phone naturally decreases and increases the distance between the stimulus and the participant (for another mobile task that operationalizes natural movements based on swipe gestures, see Meule et al., 2019).

**Fig. 2:**
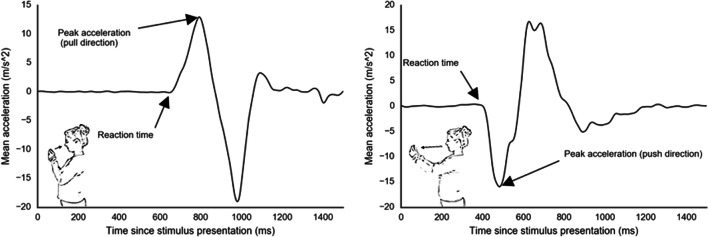
Prototypical sensor output of the mobile AAT. Accelerometer output for a prototypical approach (left) and avoidance movement (right).

Just like other AATs, the mobile AAT can detect both the direction and the reaction time of each response. It detects these features by using motion sensors to track responses. In addition to detecting approach-avoidance tendencies based on traditionally measured reaction times, using motion sensors also allows for detection of tendencies based on other response features, such as response force (see Fig. 2). Response force has recently been shown to be closely related to motivation strength in humans (Yoon, Geary, Ahmed, & Shadmehr, 2018) and is traditionally used to measure approach-avoidance motivation in animals (Brown, 1948; da Silva, Tecuapetla, Paixão, & Costa, 2018; Evans et al., 2018; Niv, Daw, Joel, & Dayan, 2007). However, although researchers have used custom-built equipment to measure force-related approach-avoidance effects, to our knowledge, none have yet succeeded in detecting them (Puca, Rinkenauer, & Breidenstein, 2006; Rotteveel & Phaf, 2004; Solarz, 1960).

## Current research

In two experiments, we tested the mobile AAT by replicating an established approach-avoidance effect toward happy and angry faces (Marsh, Ambady, & Kleck, 2005; Roelofs, Minelli, Mars, van Peer, & Toni, 2009; Rotteveel & Phaf, 2004; Seidel, Habel, Kirschner, Gur, & Derntl, 2010; Stins et al., 2011; Veenstra, Schneider, Bushman, & Koole, 2017). In general, people react quicker when instructed to approach happy or avoid angry faces (congruent trials) compared to when these instructions are reversed (incongruent trials). In addition to this well-established approach-avoidance effect, we hypothesized that people react with greater response force in congruent compared to incongruent trials. We tested the occurrence of these effects in two experiments. Experiment 1 (not preregistered) served as a pilot, in which we aimed to replicate the classical (reaction time-based) effect and, for the first time, to demonstrate response force-based effects. In Experiment 2, we replicated Experiment 1 in a preregistered field study, including a larger and more diverse sample. To explain some of the between-participant variation in approach-avoidance effects discovered in Experiment 1, we also included an additional explanatory variable (trait anger), which has previously been shown to moderate approach-avoidance effects toward happy and angry faces (Veenstra et al., 2017).

## Experiment 1

### Method

#### Participants

In Experiment 1, 64 female students from Leiden University participated in exchange for a monetary reward (€4.50) or course credit. The sample size was determined by a power analysis (G*Power; Faul, Erdfelder, Lang, & Buchner, 2007) with an effect size of g = 0.34 (based on a meta-analysis by Phaf, Mohr, Rotteveel, & Wicherts, 2014) and a power of 90%. Only female participants were chosen both to reduce noise due to gender differences and because earlier research has suggested that women have more pronounced approach-avoidance tendencies than men (Rotteveel & Phaf, 2004; Solarz, 1960). Nine participants had to be excluded because of too few valid trials either in the mobile or in the computerized AAT (for criteria, see the data exclusions section, preregistration, and the project’s Open Science Framework page; https://osf.io/y5b32/). A follow-up analysis indicated that some of these exclusions were due to a sensor error which caused implausibly short reaction times in the mobile AAT (see the project’s Open Science Framework page; https://osf.io/y5b32/). The final sample of Experiment 1 included 55 participants between the age of 18 and 29 years (*M* = 21.6, *SD* = 2.6). The study was approved by the institutional ethics board (3832757848) and informed consent was obtained from all participants.

#### Procedure.

After filling in the informed consent, half of the participants first completed the mobile AAT and half first completed the joystick AAT. Instructions on how to complete each AAT were given verbally by the experimenter. Participants were instructed to stand during the mobile AAT. The experimenter remained in the room during the practice trials to ensure all movements were performed correctly, but left the room during the experimental trials. After the first AAT, participants completed the filler task before completing the second AAT. Finally, they completed the stimulus rating task, were debriefed, and rewarded for their participation.

#### Mobile AAT

In Experiment 1, the mobile AAT was completed on an iPhone 3GS provided by the experimenter. This first version of the mobile AAT was programmed in Objective-C using Xcode, which made it usable only on iOS devices (note, however, that the iPhone version of the mobile AAT is currently no longer developed as we switched to Android to speed up deployments; see Experiment 2). During the mobile AAT, pictures of happy and angry faces were presented on a smartphone that participants were instructed to either pull toward themselves or push away from themselves. Participants completed two blocks—a congruent and an incongruent one. The order in which these blocks were completed was counterbalanced between participants. In the congruent block, they were instructed to pull happy faces toward themselves and to push angry faces away from themselves. In the incongruent block, the instructions were reversed. This means that participants were instructed to attend to the stimulus feature, based on which the approach-avoidance effect was later calculated (feature-relevant instructions). During each block, five happy and five angry face stimuli (taken from Rotteveel & Phaf, 2004) were presented four times each (repeated within but not between blocks), yielding a total of 80 trials. Throughout the task, participants were instructed to hold the phone in a horizontal orientation and, between trials, to move the phone to a starting position from which they could easily pull it toward themselves or push it away from themselves (see Fig. 1). Before each block, they were instructed which stimuli to pull and which to push. They were also instructed to react as quickly and accurately as possible. Each stimulus was preceded by a fixation cross, which remained on screen for 1.5 seconds. During each response, the phone’s accelerometers and gyroscopes tracked the gravity- and rotation-corrected acceleration of the movement in the direction perpendicular to the face of the screen (100Hz sampling rate). Based on this acceleration the responses, reaction times, and response forces were calculated (see Fig. 2). If no response was given within two seconds, a clock was displayed on the screen to inform participants that the trial had timed out. Before each block, participants were presented with an additional ten practice trials, which unlike experimental trials were followed by a response feedback (a green screen for a correct response and a red screen for an incorrect response). The source code of the mobile AAT is available on the project’s Open Science Framework page (https://osf.io/y5b32/).

#### Control tasks

##### Joystick AAT

To investigate whether our design could successfully evoke approach-avoidance tendencies, participants also completed a computerized version of the AAT (the joystick AAT; (Wiers, Rinck, Dictus, & van den Wildenberg, 2009). The joystick AAT was similar to the mobile AAT except that participants were presented with stimuli on a laptop screen and instructed to approach or avoid these by pulling or pushing a joystick. Approach was simulated by increasing the stimulus size during pull movements and avoidance was simulated by decreasing the stimulus size during push movements (Rinck & Becker, 2007). Also, as is common practice in joystick AATs, reaction times were recorded at 30% of the maximum joystick extension. To reduce learning effects, stimuli were not repeated between the two AATs, but their presentation was counterbalanced so that, across the whole sample, each stimulus appeared equally often in the mobile and in the joystick AAT. To further reduce learning effects, the two AATs were separated by an unrelated filler task (an associative priming lexical decision task; de Groot, 1984; Matzke et al., 2015).

##### Stimulus ratings

As an additional manipulation check, we included a stimulus rating task in which participants were asked to rate each stimulus’s valence and approachability on seven-point scales ranging from 1 = “not positive/approachable at all” to 7 = “very positive/approachable”.

##### Data preprocessing

To detect the direction, reaction time (RT), and response force (RF) of each reaction, we analyzed the acceleration of the phone in the direction perpendicular to the ventral axis of the participant (see Fig. 2). Raw acceleration measures were first interpolated. Next, the first peak was detected based on a zero-crossing derivative, with the condition that the amplitude of detected peaks should be at least 30% of the maximum amplitude of the response and that peaks should be at least 10 ms apart from each other. These cutoffs were chosen based on visual inspection of a random selection of trials and preregistered in Experiment 2. Responses were categorized based on the sign of the first peak (an initial positive peak indicates an approach response, whereas an initial negative peak indicates an avoidance response). Next, RTs were detected based on an absolute acceleration cutoff of 0.5 meters per second squared (m/s^2) on the left side of the first peak. This cutoff was chosen based on visual inspection of a random sample of response curves. We chose this cutoff to get to the earliest detectible change in acceleration, while at the same time preventing the algorithm from picking up sensor noise as responses. The cutoff was preregistered for Experiment 2. To detect RFs, we used the magnitude of the first acceleration peak as a proxy. As the mass of the phone was constant throughout the experiment, force should be directly related to acceleration and all standardized differences in acceleration should exactly reflect standardized differences in force. Data preprocessing was performed using Python (version 3.5.5). All preprocessing scripts are available on the project’s Open Science Framework page (https://osf.io/y5b32/).

##### Data exclusions

Practice trials, error trials, trials with missing sensor data, trials with implausibly short reaction times (< 200 ms), and trials with low absolute maximum forces (< 1 m/s^2; indicating non-responses) were excluded before analysis. Participants with less than 80% valid experimental trials were also excluded. All data, including those of excluded participants, are available on the project’s Open Science Framework page (https://osf.io/y5b32/).

##### Analysis strategy

Statistical analyses were performed using R (version 3.4.3). Following recommendations of Baayen and Milin (2010), we analyzed our data using linear mixed effects models (LMMs; using the lmerTest package). LMMs have the advantage over more commonly used repeated measures ANOVAs that they do not require data aggregation and avoid the resulting loss of information. The primary effects of interest in this study were the interaction effects between response direction (approach vs. avoidance) and stimulus type (happy vs. angry) on RT and RF. Because RTs tend to be non-normally distributed, they need to either be transformed or analyzed using generalized LMMs. In our analyses, we used the inverse transformation (1/RT) to normalize RTs while at the same time keeping values interpretable. Inverted RTs can be interpreted as the number of reactions a participant can perform in one second. We followed recommendations by Pek and Flora (2018) and reported only unstandardized effect sizes, which means that all estimated mean RTs and RT effect sizes were reported in reactions per second (1/s) and all estimated mean RFs and RF effect sizes were reported in meters per second squared (m/s^2). Initial model comparisons based on data from Experiment 1 indicated that both by-participant random intercepts and by-participant random slopes were best supported by our data. These comparisons were based on Aikake Information Criteria (Akaike, 1998; Matuschek, Kliegl, Vasishth, Baayen, & Bates, 2017). Including random slopes has the advantage that it increases generalizability compared to including only random intercepts (Barr, Levy, Scheepers, & Tily, 2013) and also allows us to calculate the split-half reliabilities of approach-avoidance effects based on mixed models. To calculate split-half reliabilities, we split each dataset into odd and even trials (balanced by response direction and stimulus type) and ran a separate mixed model for each split. We then extracted the resulting by-participant random slopes for each model and correlated them. Finally, we applied the Spearman-Brown correction to the correlations to acquire split-half reliabilities. The necessity of random slopes also indicates the presence of possibly explainable between-participant variance. To investigate this possibility, we added between-participant moderator variables in Experiment 2. To decrease collinearity and to allow us to interpret simple effects as main effects, we dummy coded response direction (***is_pull***) and stimulus type (***is_happy***) and mean-centered all predictor variables. The complete models tested in Experiment 1 were defined as:

$$ \boldsymbol{1}/\boldsymbol{RT}\sim \boldsymbol{is}\_\boldsymbol{pull}\ast \boldsymbol{is}\_\boldsymbol{happy}+\left(\boldsymbol{1}+\boldsymbol{is}\_\boldsymbol{pull}\ast \boldsymbol{is}\_\boldsymbol{happy}\ |\ \boldsymbol{participant}\right)\boldsymbol{force}\sim \boldsymbol{is}\_\boldsymbol{pull}\ast \boldsymbol{is}\_\boldsymbol{happy}+\left(\boldsymbol{1}+\boldsymbol{is}\_\boldsymbol{pull}\ast \boldsymbol{is}\_\boldsymbol{happy}\ |\ \boldsymbol{participant}\right) $$

All analysis scripts, including additional robustness checks, analyses with modeled autocorrelation, and analyses of response accuracy/error data are available on the project’s Open Science Framework page (https://osf.io/y5b32/).

### Results

#### Mobile AAT

**Fig. 3:**
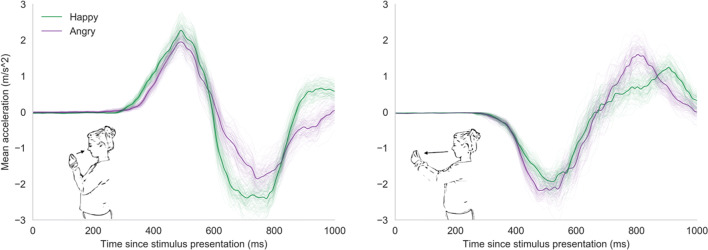
Average sensor output per cell in Experiment 1. This figure shows the average raw sensor output for approach movements (left) and avoidance movements (right) for happy and angry faces across all analyzed trials. The y-axis represents the mean acceleration. Positive values indicate acceleration of the phone toward the participant (approach) and negative values acceleration of the phone away from the participant (avoidance). The x-axis represents the time in milliseconds (ms) since stimulus presentation. The thin lines represent average accelerations from 100 bootstrapped samples. It can be seen that the average approach response is more pronounced and is initiated quicker for happy than for angry faces. The reverse effect can be seen in the average avoidance response.

**Fig. 4:**
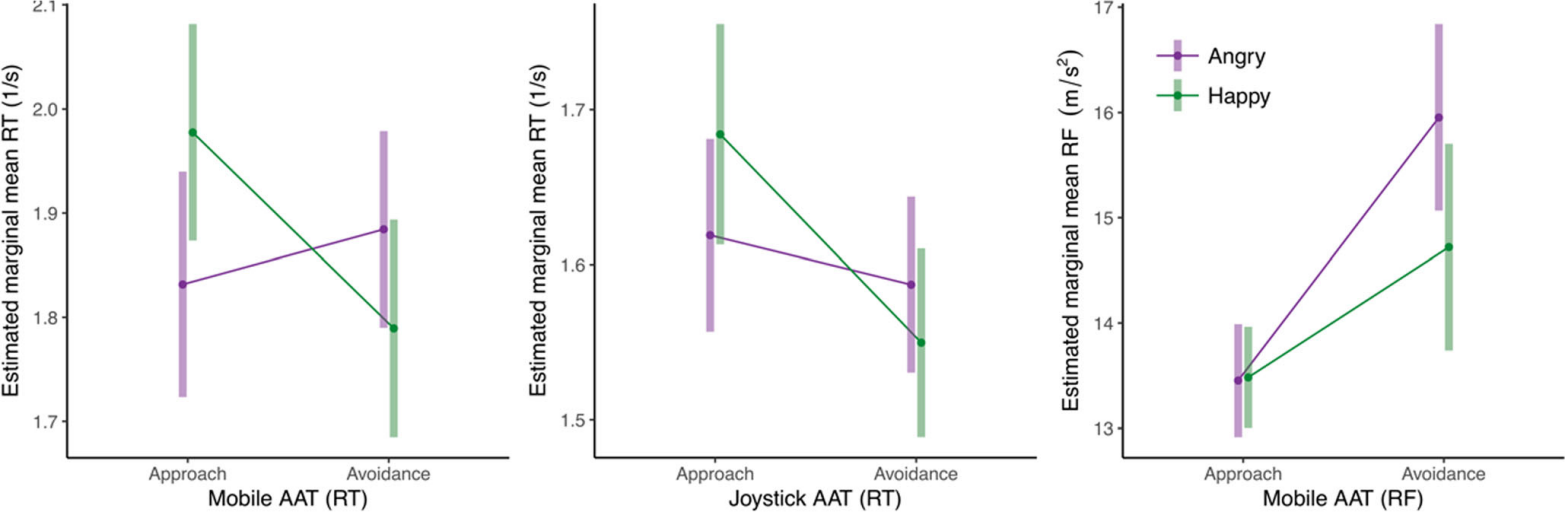
Estimated means Experiment 1. Estimated mean inverted RT (1/s) for mobile AAT (left) and joystick AAT (middle) and estimated mean RF for mobile AAT (right) for approach and avoidance responses toward happy and angry faces.

Of the experimental trials, 7.2% were excluded from the analysis (for criteria, see the data exclusions section, preregistration and the project’s Open Science Framework page; https://osf.io/y5b32/). We analyzed the mobile AAT data in two separate mixed models, one with inverted RT and one with RF as the dependent variable. In the RT mixed model, there was a significant main effect of response direction (*b* = 0.068 [0.032, 0.118], *t* = 3.09, *p* = .003) as participants, generally, reacted faster when approaching (*M* = 1.90, *SE* = 0.052) compared to avoiding stimuli (*M* = 1.84, *SE* = 0.049). There was no main effect of stimulus type. More importantly, there was a significant interaction effect between response direction and stimulus type on RT (*b* = 0.241 [0.138, 0.346], *t* = 4.61, *p* <.001). As hypothesized, participants reacted faster when approaching happy (*M* = 1.98, *SE* = 0.053) compared to angry faces (*M* = 1.83, *SE* = 0.055), and reacted faster when avoiding angry faces (*M* = 1.88, *SE* = 0.048) compared to happy faces (*M* = 1.79, *SE* = 0.053; see Figs. 3 and 4). In the RF mixed model, there was a significant main effect of response direction (*b* = -1.868 [-2.615, -1.084], *t* = -5.00, *p* < .001), as well as a main effect of stimulus type (*b* = -0.601 [-0.930, -0.306], *t* = -3.55, *p* = .001) on RF. On average, participants used less force in approach movements (*M* = 13.47, *SE* = 0.236) compared to avoidance movements (*M* = 15.34, *SE* = 0.443) and less force when reacting to happy faces (*M* = 14.10, *SE* = 0.319) compared to angry faces (*M* = 14.70, *SE* = 0.308). More importantly, there was a significant interaction between response direction and stimulus type on RF (*b* = 1.262 [0.438, 2.207], *t* = 2.62, *p* = .012; see Figs. 3 and 4). As hypothesized, participants used more force to approach happy faces (*M* = 14.72, *SE* = 0.501) compared to angry faces (*M* = 13.45, *SE* = 0.274) and more force to avoid angry faces (*M* = 15.95, *SE* = 0.452) compared to happy faces (*M* = 14.72, *SE* = 0.501). The Spearman-Brown split-half reliability was high for both RT-based (*r* = .77) and RF-based approach-avoidance effects (*r* = .84).

#### Control tasks

##### Joystick AAT

Of the experimental trials, 7.0% were excluded from the analysis (for criteria, see the preregistration and the project’s Open Science Framework page; https://osf.io/y5b32/). In the joystick AAT, there was a significant main effect of response direction on RT (*b* = 0.083 [0.057, 0.110], *t* = 7.10, *p* < .001) as participants, generally, reacted faster when approaching (*M* = 1.65, *SE* = 0.031) compared to avoiding (*M* = 1.57, *SE* = 0.027) stimuli. There was no main effect of stimulus type on RT. More importantly, there was a significant interaction effect between response direction and stimulus type on RT (*b* = 0.102 [-0.065, 0.260], *t* = 2.14, *p* = .037). As hypothesized, participants initiated approach responses toward happy faces faster (*M* = 1.68, *SE* = 0.036) than toward angry faces (*M* = 1.62, *SE* = 0.032), and they initiated avoidance responses toward angry faces faster (*M* = 1.59, *SE* = 0.029) than toward happy faces (*M* = 1.55, *SE* = 0.031; see Fig. 4). The Spearman-Brown split-half reliability of the approach-avoidance effect was high (*r* = .81).

##### Stimulus ratings

To confirm that happy faces were perceived as more positive and more approachable than angry faces, we performed two mixed model analyses with stimulus type as predictor and valence and approachability ratings as outcome variables. Results of these analyses indicated that stimulus type indeed successfully predicted valence (*b* = 4.064 [3.875, 4.259], *t* = 40.48, *p* < .001) and approachability ratings (*b* = 4.010 [3.875, 4.259], *t* = 31.16, *p* < .001), with happy faces being rated both as more positive (*M* = 6.80, *SE* = 0.095) and more approachable (*M* = 6.61, *SE* = 0.137) than angry faces (valence: *M* = 2.74, *SE* = 0.101; approachability: *M* = 2.60, *SE* = 0.111).

##### Comparison of effect sizes

The analyses of the mobile and joystick data indicated that the approach-avoidance effect detected by the mobile AAT was larger than that detected by the joystick AAT. To test this difference in an exploratory analysis, we added task type (mobile vs. joystick) to the RT mixed model. The resulting three-way interaction between task type, response direction, and stimulus type was indeed significant (*b* = 0.133 [-0.005, 0.281], *t* = 3.92, *p* < .001), confirming that the approach-avoidance effect detected by the mobile AAT was larger than that detected by the joystick AAT.

##### Correlation between effects

In another exploratory analysis, we tested the correlations between RT-based approach-avoidance effects in the mobile AAT, RF-based approach-avoidance effects in the mobile AAT, and RT-based approach-avoidance effects in the joystick AAT. To do so, we extracted random slopes for each participant from each of the models and correlated these random slopes. None of these correlations were significant (*r*s < .05, *p*s > .800; see discussion).

### Discussion

Together, the results of Experiment 1 indicate that the mobile AAT successfully detected approach-avoidance effects, based both on reaction times and response forces. As predicted, participants reacted faster and with more force when having to approach happy or avoid angry faces compared to when they had to avoid happy or approach angry faces. In an exploratory analysis, we found no correlation between effects detected by the mobile and the joystick AAT. This lack of association is surprising, but others have likewise not observed such a correlation (Krieglmeyer & Deutsch, 2010). We also found no correlation between RT-based and RF-based approach-avoidance effects in the mobile AAT. Although this could mean that the two effects are driven by separate processes, further research is necessary to test this hypothesis. In a final exploratory analysis, we found larger approach-avoidance effects with the mobile compared to the joystick AAT. This finding indicates that the mobile AAT might be more sensitive than other versions of the AAT, although this too requires further examination.

In summary, Experiment 1 provided a promising first test of the mobile AAT. Yet, Experiment 1 also had some limitations. First, it was based on a relatively small sample of only female participants. Second, although one of the main advantages of the mobile AAT is that it can easily be deployed in the field, Experiment 1 tested it in the laboratory. Finally, although model comparison indicated between-participant differences that might be explained by between-participant variables, no such variables were included in Experiment 1. Experiment 2 addresses these limitations.

## Experiment 2

In Experiment 2, we tested the mobile AAT in a preregistered field study using a larger and more diverse sample than in Experiment 1 (for preregistration, see the project’s Open Science Framework page on https://osf.io/kmh4d). The main purpose of Experiment 2 was to replicate and further generalize findings of Experiment 1, by including both women and men in the sample and by testing the mobile AAT’s usability in the field. In addition, as model comparison in Experiment 1 indicated significant between-participant differences in approach-avoidance effects, we included an exploratory variable—trait anger—to explain some of these differences. Trait anger has recently been demonstrated to moderate and even reverse approach-avoidance effects toward happy and angry faces (Veenstra et al., 2017). This effect is especially interesting as it is one of the few examples in which participants tend to approach negative and avoid positive stimuli. We therefore, in addition to the hypotheses of Experiment 1, included two extra hypotheses, predicting that with increasing trait anger both RT-based and RF-based approach-avoidance effects would decrease.

### Method

#### Participants

A power analysis based on Experiment 1 indicated that a sample of 150 participants would allow us to detect approach-avoidance effects based on RT (*b* = 0.241) and RF (*b* = 1.262) with a power of 99% (for method used, see Brysbaert & Stevens, 2018, and the project’s Open Science Framework page; https://osf.io/y5b32/). Participants were US citizens recruited via the online recruitment platform Prolific Academic (www.prolific.co). Excluded participants (for criteria, preregistration, and the project’s Open Science Framework page; https://osf.io/y5b32/) were replaced by additional participants until the preregistered sample size was reached. In total, 195 participants participated in the experiment, 44 of which had to be excluded because of too many invalid trials (for a detailed analysis of exclusion reasons, see the the project’s Open Science Framework page; https://osf.io/y5b32/). The final sample consisted of 151 participants (76 women). Participants’ ages ranged from 18 to 37 years (*M* = 27.07, *SD* = 5.07). The study was approved by the institutional ethics board (CEP18-0705/300) and informed consent was obtained from all participants.

#### Procedure

Participants were instructed to download the mobile AAT application to their smartphone by clicking on a link in Prolific Academic (prolific.co). All instructions were given and all tasks were completed within this application. After providing informed consent, participants filled in their age and gender and completed the mobile AAT. Next, they filled in the trait anger questionnaire, were debriefed, and rewarded for their participation.

#### Mobile AAT

The mobile AAT app used in Experiment 2 resembled that used in Experiment 1. However, instead of running on iOS, this version of the task ran on Android (accordingly the task was programmed in Java using Android Studio). Android was preferred over iOS as Android applications are easier and quicker to deploy than those programmed for iOS, which facilitates prototyping. To further ease the use of the app, it was programmed so that new experiments could be generated without altering the source code (see the project’s Open Science Framework page; https://osf.io/y5b32/ for an example experiment). Instead of using the experimenter's phone, as in Experiment 1, in Experiment 2, participants downloaded the AAT as an application and completed the AAT on their own smartphones. Instead of being instructed by the experimenter, as in Experiment 1, they were instructed in the application. In these instructions, correct movements were displayed in an HTML file in the application which included two GIF animations, displaying examples of approach and avoidance movements (see the project’s Open Science Framework page; https://osf.io/y5b32/). A final difference to the AAT used in Experiment 1 was that participants completed 120 instead of 80 trials, as each stimulus was repeated six instead of four times.

#### Trait anger questionnaire

We measured trait anger with the trait dimension of the State-Trait Anger Scale (STAI; Clausen et al., 2016; Spielberger, Jacobs, & Russell, 1983; Veenstra et al., 2017). The trait dimension of the STAI consists of ten statements (e.g., “I am quick tempered”) for each of which participants indicate how they generally feel or react. In this experiment, participants responded to each item by selecting one out of four multiple choice options. Before analysis, each of these options was assigned an ordinal number, which—to ensure comparability with earlier studies—was projected on a range from 1 to 100 (i.e., 1 = “*Almost never*”, 34 = “*Sometimes*”, 67 = “*Often*”, 100 = “*Always*”). Trait anger scores were calculated by taking the mean of all responses for each participant.

#### Data analysis strategy

We followed the same data analysis strategy as in Experiment 1. To also analyze the effect of mean trait anger, we added the centered mean trait anger scores (***trait_anger***) to the mixed models, resulting in the following mixed models:

$$ \boldsymbol{1}/\boldsymbol{RT}\sim \boldsymbol{is}\_\boldsymbol{pull}\ast \boldsymbol{is}\_\boldsymbol{happy}+\left(\boldsymbol{1}+\boldsymbol{is}\_\boldsymbol{pull}\ast \boldsymbol{is}\_\boldsymbol{happy}\ |\ \boldsymbol{participant}\right)\boldsymbol{force}\sim \boldsymbol{is}\_\boldsymbol{pull}\ast \boldsymbol{is}\_\boldsymbol{happy}\ast \boldsymbol{trait}\_\boldsymbol{anger}+\left(\boldsymbol{1}+\boldsymbol{is}\_\boldsymbol{pull}\ast \boldsymbol{is}\_\boldsymbol{happy}\ |\ \boldsymbol{participant}\right) $$

### Results

#### Confirmatory analyses

**Fig. 5:**
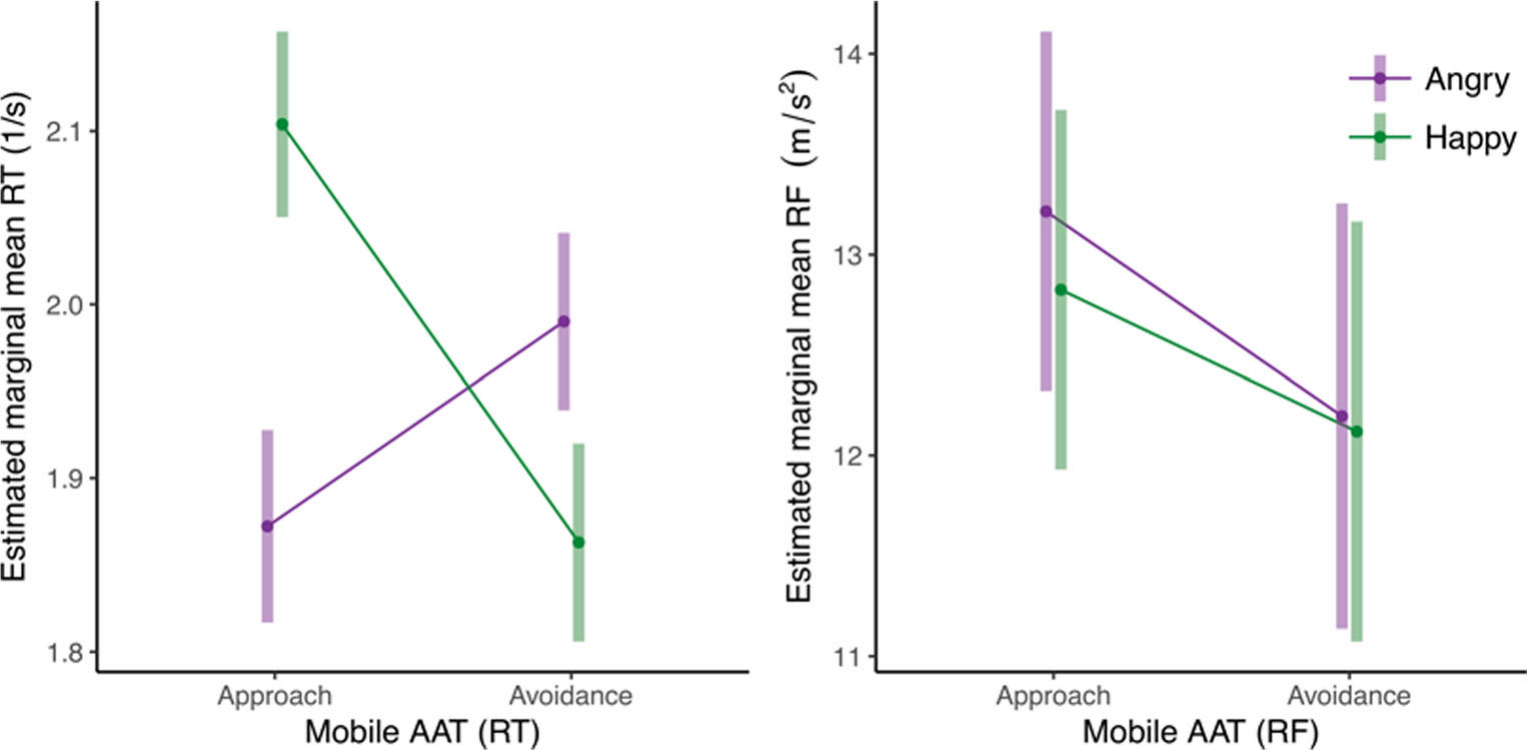
Estimated means for Experiment 2. Estimated mean inverted RT (1/s; left) and RF (right).

**Fig. 6:**
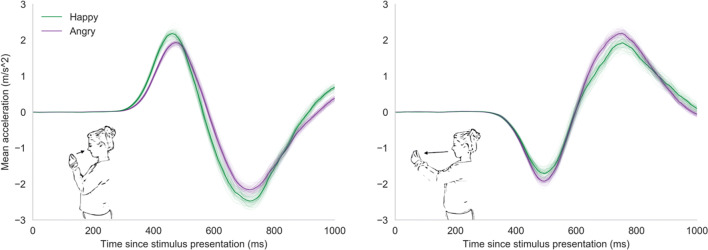
Average sensor output per cell in Experiment 2. This figure shows the average raw sensor output for approach movements (left) and avoidance movements (right) for happy and angry faces across all analyzed trials. The y-axis represents the mean acceleration. Positive values indicate acceleration of the phone toward the participant (approach) and negative values acceleration of the phone away from the participant (avoidance). The x-axis represents the time in milliseconds (ms) since stimulus presentation. The thin lines represent average accelerations from 100 bootstrapped samples. It can be seen that the average approach response is more pronounced and is initiated quicker for happy than for angry faces. The reverse effect can be seen in the average avoidance response.

Of the experimental trials, 8.6% were excluded from the analysis (for criteria, preregistration, and the project’s Open Science Framework page; https://osf.io/y5b32/). Similar to Experiment 1, there was a significant main effect of response direction on RT (*b* = 0.061 [0.045, 0.079], *t* = 7.26, *p* < .001), as well as a main effect of stimulus type (*b* = 0.052 [0.036, 0.070], *t* = 5.81, *p* < .001) on RT. On average, participants were faster to approach stimuli (*M* = 1.99, *SE* = 0.026) than to avoid them (*M* = 1.93, *SE* = 0.025). Participants also reacted faster toward happy faces (*M* = 1.98, *SE* = 0.026) compared to angry faces (*M* = 1.93, *SE* = 0.025). More importantly, there was a significant interaction between response direction and stimulus type on RT (*b* = 0.359 [0.272, 0.429], *t* = 8.75, *p* < .001). As hypothesized, participants were faster to approach happy faces (*M* = 2.10, *SE* = 0.027) compared to angry faces (*M* = 1.87, *SE* = 0.028) and faster to avoid angry faces (*M* = 1.99, *SE* = 0.026) compared to happy faces (*M* = 1.86, *SE* = 0.029; see Figs. 5 and 6).

Similar to Experiment 1, there was a significant main effect of response direction on RF (*b* = 0.863 [0.342, 1.382], *t* = 3.16, *p* = .002), as well as a main effect of stimulus type (*b* = -0.234 [-0.495, -0.007], *t* = -1.98, *p* = .049) on RF. As in Experiment 1, participants used more force when reacting to angry faces (*M* = 12.71, *SE* = 0.460) compared to happy faces (*M* = 12.47, *SE* = 0.451). However, in contrast to Experiment 1, participants used more force in approach movements (*M* = 13.02, *SE* = 0.431) than in avoidance movements (*M* = 12.16, *SE* = 0.510; see Fig. 5). Counter to our predictions, there was no significant interaction effect between response direction and stimulus type (*b* = -0.315 [-1.466, 0.766], *t* = -0.53, *p* = .600). Spearman-Brown split-half reliability analyses indicated high reliability of both RT-based (*r* = .91) and force-based approach-avoidance effects (*r* = .97).

Counter to our predictions, there were no three-way interactions between response direction, stimulus type, and trait anger (RT: *b =* -0.002 [-0.008, 0.003], *t* = -0.90, *p =* .372; RF: *b =* -0.040 [-0.112, 0.018], *t =* -1.00, *p =*.317). Participants' trait anger scores ranged from 1 to 70 (*M* = 27.66; *SD =* 15.03).

#### Exploratory analyses

Although some authors have suggested that female participants show larger approach-avoidance effects than male participants (Rotteveel & Phaf, 2004; Solarz, 1960) to our knowledge, no study has, so far, shown this to be the case. To test this effect, we analyzed the interaction between response direction, stimulus type, and gender. This interaction was, indeed, significant (*b* = -0.208 [-0.359, -0.045], *t* = 3.16, *p* = .002). To determine the direction of the effect, we analyzed the data separately for female and male participants. These analyses confirmed that female participants showed larger approach-avoidance effects toward happy and angry faces (*b* = 0.462 [0.335, 0.569]) than male participants (*b* = 0.254 [0.124, 0.375]).

## General Discussion

### Summary of results

We tested a mobile version of the AAT by replicating a well-established approach-avoidance effect toward happy and angry faces. Participants approached and avoided pictures of happy and angry faces, by moving a smartphone toward or away from themselves, while their reaction times (RT) and response forces (RF) were measured. We expected that participants would be faster and use more force to approach happy and avoid angry faces, compared to when these instructions were reversed. Our results indicate that the mobile AAT was, indeed, able to reliably detect this effect on RT, both in the laboratory (Experiment 1) and in the field (Experiment 2). The detected effects were larger than those detected by a traditional version of the AAT (i.e., the joystick AAT) and the split-half reliability was high. Effects, however, did not correlate with those detected by the joystick AAT. In addition to detecting the classical RT-based approach-avoidance effect, we also assessed the existence of an approach-avoidance effect based on the novel measurement of RF. We successfully detected this effect in the laboratory (Experiment 1) but were not able to replicate it in the field (Experiment 2). Nevertheless, split-half reliabilities of force-based effects were high in both experiments. Because model comparisons pointed to considerable between-participant variability, we also included a trait-anger measure in our second experiment to test whether participants with increased trait anger scores show decreased or even reversed approach-avoidance effects toward happy and angry faces. This proved not to be the case, in our study.

### Limitations

Although the mobile AAT reliably detected the hypothesized approach-avoidance effect, this effect did not correlate with that measured by the joystick AAT. This might indicate that the mobile AAT did not tap into the same processes as the joystick AAT. The similarities and differences between different versions of the AATs are not well understood and, at this point, it is not known whether different versions of the AAT actually measure the same processes. As noted, the one study comparing different versions of the AAT (the joystick and a manikin version; Krieglmeyer & Deutsch, 2010) similarly did not find a correlation between tasks. In line with the idea that different AATs might tap into different processes, Krieglmeyer and Deutsch (2010) suggested that in the joystick AAT “participants […] might represent their behavior as merely increasing or decreasing the size of the stimulus, which is unrelated to approach-avoidance” (p. 814). Another reason for a lack of correlation might be a potential low test-retest reliability of either or both of the tasks. The only study which, to our knowledge, assessed the test-retest reliability of the joystick AAT indeed reports a low reliability (r = .35, Reinecke, Soltau, Hoyer, Becker, & Rinck, 2012).

Although we did find an RF-based approach-avoidance effect in the laboratory, we were not able to replicate this effect in the field. This might be due to the lack of control that field experiments inevitably bring with them. It is possible that participants did not perform prototypical approach-avoidance movements, which might have led to increased noise in the force measurements (note that RT measurements should be less affected by the exact execution of a response, as these depend only on the response’s initiation time). A further source of noise could be the variety of devices used in Experiment 2. As there are hundreds of Android devices built by dozens of companies, it is not certain that all devices detect force with the same sensitivity and precision. Future research with the mobile AAT should therefore log the device type, so that this information can be added to statistical models (this feature is already implemented in the newest version of the application). Next to increased noise, there might also be systematic differences between Experiment 1 and Experiment 2, as it is noteworthy that the main effect of response direction was reversed in Experiment 2. This difference in main effects could, for example, be caused by differences in instructions, as participants were instructed to stand in Experiment 1, but received no such instruction in Experiment 2. It should be mentioned that, although the lack of correlation between RT-based and force-based approach-avoidance biases is surprising, it is not implausible. In our design, RTs are measured at the very beginning of a response, that is, before force has a significant influence on it. This means that higher response forces do not necessarily lead to shorter RTs. Therefore, a lack of correlation between the two measures might indicate that the two aspects of the response could be driven by different processes (although future studies would need to investigate this more specifically).

We were not able to replicate findings by Veenstra et al. (2017) who observed reversed RT-based approach-avoidance effects in participants high in trait anger. The effect found by Veenstra et al. (2017) is theoretically relevant as it is, to our knowledge, the only known finding in which RT-based approach-avoidance tendencies dissociate from valence (i.e., usually participants approach positive and avoid negative stimuli). However, the effect is far from established as a study by Clausen et al. (2016), for example, did not yield reversed effects in war veterans high in trait anger (although see Harmon-Jones & Gable, 2018, for dissociations between valence and neurological markers of approach-avoidance motivations). Both Clausen et al. (2016) and our second experiment used an American sample, whereas Veenstra et al. (2017) used a Dutch sample. It is therefore also possible that cultural differences influence whether participants high in trait anger approach rather than avoid angry faces.

The main purpose of this study was to test the mobile AAT’s ability to detect established approach-avoidance effects. Therefore, we designed the study to maximize effects, so that possible null-findings could be fully attributed to problems with the task (rather than to the design). Other designs are possible, which might explain inconsistencies between our findings and findings of other studies. For example, in our studies we used feature-relevant instructions, which means that participants were instructed to respond to the stimulus dimension that was also used to calculate approach-avoidance tendencies. A relevant feature design was chosen to maximize effects, although irrelevant feature designs, in which participants are not made aware of the measured stimulus dimension, are more implicit (Phaf, Mohr, Rotteveel, & Wicherts, 2014). Another choice we made to maximize effects was to not include a neutral stimulus condition. This design choice, however, has the limitation that it does not allow us to interpret the individual contribution of effects within each movement (approach and avoidance) to the approach-avoidance effect. It also makes it difficult to interpret possible asymmetries in the task that could influence the interpretation of interaction effects. The purpose of this study was to show the feasibility of the mobile AAT in a first test. Future studies could further test the task by including irrelevant feature instructions and neutral stimuli.

We designed the mobile AAT to provide a task that is easily used in the field and allows for natural approach and avoidance movements. While these are advantages of the mobile AAT, implementing them required certain design choices that come with certain limitations. For example, the mobile AAT relies only on arm movements. Virtual reality-based AATs implement full-body movements that might capture distinct effects (e.g., Eder, Krishna, Sebald, & Kunde, 2019). However, these tasks are (at the time of writing) also less mobile than the mobile AAT, as they require specialized and stationary equipment. Another design choice was to allow participants in the mobile AAT to change the distance between themselves and the stimulus using natural movements. This has the advantage of naturally disambiguating responses without relying on virtual effects. However, if the purpose of a study is to disentangle movements from distance change, tasks that allow for such virtual effects are required (e.g., Markman & Brendl, 2005).

Finally, it should be mentioned that other mobile tasks have been recently suggested, each distinct from our version of the mobile AAT. For example, Kakoschke and colleagues (2018) developed a smartphone-based version of the AAT in which participants tilt the phone to approach or avoid stimuli. Tilting the phone involves less work than actually moving the phone away or towards oneself as in the mobile AAT. However, no distance is changed while tilting the phone, and, at the time of writing, this version of the AAT does not measure reaction times and can therefore only be used for cognitive bias modification therapy. Another mobile AAT has recently been developed by Meule et al. (2019). In this AAT, participants swipe stimuli towards or away from themselves on a horizontally oriented touchscreen. The task is therefore also mobile, and also allows participants to vary the actual distance to the stimulus. This operationalization is, however, somewhat more complex than that of the mobile AAT presented here, as approach and avoidance movements entail two stages: In the first stage participants move their hand from a resting position toward the stimulus; in the second stage, they move the stimulus toward or away from themselves. Whether the above listed differences between the mobile AAT and other mobile tasks influence approach-avoidance tendencies, could be assessed in future empirical studies.

### Implications

We showed that the mobile AAT can successfully measure well-established approach-avoidance tendencies in the laboratory and in the field, with both high sensitivity and high reliability. Future studies can therefore use the mobile AAT to address questions which were, to date, difficult to test with existing AATs. It is, for example, likely that approach-avoidance tendencies fluctuate as a function of changing desires, need states, and other contextual factors (Corr, 2013, p. 286; Eder et al., 2013; Elliot, Eder, & Harmon-Jones, 2013, p. 310; Gawronski & Brannon, 2017, p. 15; Hofmann et al., 2012; Lewin, 1936, p. 166). Yet, at this point, the few studies that have investigated how approach tendencies fluctuate as a function of changing need states have yielded mixed results and have been limited to cross-sectional designs (Piqueras-Fiszman, Kraus, & Spence, 2014; Seibt, Häfner, & Deutsch, 2007). Knowing that the mobile AAT performs well in the field, future research could test the effects of need states on participants’ approach-avoidance tendencies in momentary assessment studies and longitudinal designs. The mobile AAT could also help researchers answer the inverse question, namely how stable approach-avoidance tendencies are in the absence of changing desires. Establishing the test-retest reliability of the mobile AAT is especially important, should it be used in clinical trials in which participants are diagnosed or the effectiveness of treatments is assessed (American Psychological Association, 1954).

Although we did not consistently find approach-avoidance effects based on force, we were able to measure force reliably. The ability to measure force could help further understand the motivational aspects of approach and avoidance behavior. A defining characteristic of motivation is the energization of behavior (Elliot, 2006) and response force during approach movements has been linked to motivation both in animals (Brown, 1948), and more recently also in humans (Yoon et al., 2018). In these studies, the need state of animals (Brown, 1948) or incentive value of stimuli (Yoon et al., 2018) was varied. Future studies could use the mobile AAT while manipulating participants’ need states (e.g., vary hunger levels in participants approaching and avoiding food) to further investigate potential force-based approach-avoidance effects.

A final goal of this study was to replicate findings by Veenstra et al. (2017), showing that approach-avoidance effects can be reversed in people high in trait anger. Here, we were not able to replicate Veenstra and colleagues (2017) findings, indicating that more research is necessary to firmly establish this theoretically relevant effect.

### Conclusion

In two studies, we successfully tested a new, mobile version of the approach-avoidance task that was designed to improve upon existing AATs. The mobile AAT reliably measured approach-avoidance effects based on reaction times and reliably measured response forces, in the laboratory as well as in the field. The flexibility and field-readiness of the mobile AAT could pave the way for new lines of research, examining the dynamics of approach-avoidance tendencies across time and contexts.
